# Distinctive Mediating Effects of Subcortical Structure Changes on the Relationships Between Amyloid or Vascular Changes and Cognitive Decline

**DOI:** 10.3389/fneur.2021.762251

**Published:** 2021-12-07

**Authors:** Na-Yeon Jung, Jeong-Hyeon Shin, Hee Jin Kim, Hyemin Jang, Seung Hwan Moon, Seung Joo Kim, Yeshin Kim, Soo Hyun Cho, Ko Woon Kim, Jun Pyo Kim, Young Hee Jung, Sung Tae Kim, Eun-Joo Kim, Duk L. Na, Jacob W. Vogel, Sangjin Lee, Joon-Kyung Seong, Sang Won Seo

**Affiliations:** ^1^Department of Neurology, Pusan National University Yangsan Hospital, Pusan National University School of Medicine and Research Institute for Convergence of Biomedical Science and Technology, Yangsan, South Korea; ^2^School of Biomedical Engineering, Korea University, Seoul, South Korea; ^3^Department of Neurology, Samsung Medical Center, Sungkyunkwan University School of Medicine, Seoul, South Korea; ^4^Neuroscience Center, Samsung Medical Center, Seoul, South Korea; ^5^Department of Nuclear Medicine, Samsung Medical Center, Seoul, South Korea; ^6^Department of Neurology, Gyeongsang National University School of Medicine and Gyeongsang National University Changwon Hospital, Changwon, South Korea; ^7^Department of Neurology, Kangwon National University College of Medicine, Chuncheon-si, South Korea; ^8^Department of Neurology, Chonnam National University Medical School and Hospital, Gwangju, South Korea; ^9^Department of Neurology, Chonbuk National University Medical School and Hospital, Jeonju, South Korea; ^10^Department of Neurology, Myongji Hospital, College of Medicine, Hanyang University, Goyang, South Korea; ^11^Department of Radiology, Samsung Medical Center, Seoul, South Korea; ^12^Department of Neurology, Pusan National University Hospital, Pusan National University School of Medicine, Pusan, South Korea; ^13^Montreal Neurological Institute, McGill University, Montrèal, QC, Canada; ^14^Graduate School, Department of Statistics, Pusan National University, Busan, South Korea

**Keywords:** mediation, subcortical structure, amyloid, lacune, cognition

## Abstract

**Objective:** We investigated the mediation effects of subcortical volume change in the relationship of amyloid beta (Aβ) and lacune with cognitive function in patients with mild cognitive impairment (MCI).

**Methods:** We prospectively recruited 101 patients with MCI who were followed up with neuropsychological tests, MRI, or Pittsburgh compound B (PiB) PET for 3 years. The mediation effect of subcortical structure on the association of PiB or lacunes with cognitive function was analyzed using mixed effects models.

**Results:** Volume changes in the amygdala and hippocampus partially mediated the effect of PiB changes on memory function (direct effect = −0.168/−0.175, indirect effect = −0.081/−0.077 for amygdala/hippocampus) and completely mediated the effect of PiB changes on clinical dementia rating scale sum of the box (CDR-SOB) (indirect effect = 0.082/0.116 for amygdala/hippocampus). Volume changes in the thalamus completely mediated the effect of lacune on memory, frontal executive functions, and CDR-SOB (indirect effect = −0.037, −0.056, and 0.047, respectively).

**Conclusions:** Our findings provide a better understanding of the distinct role of subcortical structures in the mediation of the relationships of amyloid or vascular changes with a decline in specific cognitive domains.

## Introduction

Alzheimer's disease (AD) and vascular dementia are the most common causes of dementia in the elderly (1). Mild cognitive impairment (MCI) is considered to be a transitional stage between normal cognition and several types of dementia. Amnestic MCI (aMCI) for Alzheimer's disease dementia and subcortical vascular MCI (svMCI) for subcortical vascular dementia (SVaD) are two examples. The amyloid beta (Aβ) burden is a characteristic pathologic marker of aMCI and AD, and lacune and white matter hyperintensities (WMH) on MRI are characteristic neuroimaging markers of svMCI and SVaD (2, 3).

Previous studies have shown that Aβ burden were associated with memory impairments, whereas SVD, including lacune and WMH, were associated with frontal executive dysfunction (4). Notably, a number of studies have focused on the mediation effects of cortical atrophy on the relationships of Aβ or SVD with cognitive impairments (5, 6). Specifically, several studies, including those from our own center, have suggested that Aβ and SVD burdens were correlated with thinning in the temporoparietal and frontal regions, which, in turn, leads to memory and frontal executive dysfunctions, respectively (6–9).

Other than the hippocampus, subcortical structures have received less attention compared with cortical atrophy. Previous studies have reported volume loss in the thalamus, striatum, and amygdala in MCI or AD (10–14). However, there have been few studies investigating the relationship between amyloid or SVD burden and atrophy in subcortical structures other than the hippocampus (15). Moreover, the longitudinal mediational effects of subcortical structure volume changes on the relationships of Aβ or SVD with cognitive impairments remain unclear. Given that the hippocampus and amygdala are responsible for memory function, these subcortical structural volume changes may mediate the relationships of Aβ with memory impairments. Likewise, increased SVD burdens are well-known to be associated with frontal executive dysfunction (2, 16). Frontal subcortical circuits, which consist of the frontal cortex, basal ganglia, and thalamus are responsible for frontal executive function (17). Therefore, it would be reasonable to expect that increased SVD burdens are associated with structural abnormalities in the basal ganglia and thalamus, which are further associated with frontal executive dysfunctions.

In the present study, we investigated whether volume changes in the hippocampus and amygdala mediate the relationships between increased Aβ uptakes and memory decline, whereas volume changes in the basal ganglia and thalamus mediate the relationship between increased SVD burdens and frontal executive dysfunction.

## Materials and Methods

### Participants

We prospectively recruited 117 patients with MCI (45 patients with aMCI and 72 patients with svMCI) from an outpatient memory clinic between September 2008 and September 2011 at Samsung Medical Center. Patients with aMCI were matched to svMCI patients' clinical dementia rating scale sum of box (CDR-SOB). Ten patients were excluded because of technical errors in MRI structural analyses (7 patients with aMCI and 3 patients with svMCI). Of the remaining 107 patients, 6 patients never returned for follow-up evaluation. The final analysis included 101 patients with MCI (34 patients with aMCI and 67 patients with svMCI) that had at least one follow-up evaluation. Patients were evaluated by clinical interview and neurological and neuropsychological examinations as previously described (18). Several experienced research neuropsychologists performed the standardized battery of neuropsychological examination, including neuropsychological tests (19), activity of daily living (ADL) scales (20), Neuropsychiatric Inventory (NPI) (21), and Clinical dementia rating (CDR). Patients with MCI were selected according to Petersen's criteria (22) with modifications (23), which are as follows: (i) a subjective cognitive complaint by the patient or his/her caregiver; (ii) normal ADL determined clinically and by the instrumental ADL scale (20); (iii) an objective cognitive decline below the 16th percentile of age and education-matched norms in at least one of four cognitive domains (language, visuospatial, memory, or frontal executive function) on neuropsychological tests described below (24); and (iv) absence of dementia. Of those who met the MCI criteria, aMCI was further diagnosed when patients showed objective memory decline below the 16th percentile (−1.0 SD) of age and education-matched norms on a verbal or visual memory test and did not have significant ischemia on MRI as described below. svMCI was further diagnosed when patients had subcortical vascular features defined as focal neurological symptoms/signs and significant ischemia on MRI. Significant ischemia was defined as WMH on T2-weighted or fluid-attenuated inversion recovery (FLAIR) images that satisfied the following criteria: (i) WMH ≥ 10 mm in the periventricular white matter (caps or rim); and (ii) WMH ≥ 25 mm (maximum diameter) in the deep white matter, consistent with an extensive white matter lesion or diffusely confluent lesion.

All patients underwent laboratory tests which included a complete blood count, blood chemistry, vitamin B12/folate, syphilis serology, and thyroid function tests. Brain MRI confirmed the absence of structural lesions, including territorial cerebral infarction, brain tumors, hippocampal sclerosis, and vascular malformation.

Written informed consent was obtained from each patient. This study was approved by the Institutional Review Board of Samsung Medical Center.

### ^11^C-PiB-PET Imaging and Data Analysis

^11^C-PiB-PET scanning was performed at Samsung Medical Center or Asan Medical Center with identical settings using a Discovery STe PET/CT scanner (GE Medical Systems). ^11^C-PiB was injected into an antecubital vein as a bolus with a mean dose of 420 MBq (range 259–550 MBq). CT scans were performed for attenuation correction 60 min after injection. The specific radioactivity of ^11^C-PiB at the time of administration was >1,500 Ci/mmol for patients and the radiochemical yield was >35%. The radiochemical purity of the tracer was >95% in all PET studies.

Data processing was performed using SPM Version 5 (SPM5) within Matlab 6.5 (MathWorks, Natick, MA). PiB-PET images were co-registered to individual MRIs. Using these parameters, MRI co-registered PiB-PET images were normalized to the MRI template. The quantitative regional values of PiB retention on the spatially normalized PiB images were obtained using an automated volume of interest analysis of 28 cortical volumes from the left and right hemispheres using the automated anatomical labeling atlas (AAL) atlas. Global PiB standard uptake value ratio (SUVR) was calculated from the volume-weighted average uptake ratio of the 28 cerebral cortical volumes of interest. The cortical target region consisted of the bilateral frontal, posterior cingulate gyri, parietal, lateral temporal, and occipital areas. Cerebral cortical uptake ratios were calculated by dividing each cortical volume of interest uptake ratio by the mean uptake of the cerebellar gray matter (cerebellum crus1 and crus2). We did not perform partial volume correction. We used PiB SUVR as a continuous variable representing the degree of amyloid-β burden.

### Neuropsychological Tests

All patients were annually evaluated using the Seoul Neuropsychological Screening Battery (SNSB) (19, 25). We assessed time-varying composite scores of memory and frontal executive functions because these two cognitive scores are well-known to be associated with amyloid and SVD burdens (26–28). We calculated composite scores of memory and frontal executive functions (24). The memory score was calculated by summing scores from verbal memory tests (Seoul Verbal Learning Test immediate recall, delayed recall, recognition score) and visual memory tests (Rey Complex Figure Test immediate recall, delayed recall, recognition score) (range: 0–144). The frontal executive score was calculated by summing scores from the category word generation test, phonemic word generation test, and the Stroop color-reading test (range: 0–55). Global cognitive function was assessed using CDR-SOB.

### Acquisition of MRI

An Achieva 3.0-Tesla MRI scanner (Philips, Best, The Netherlands) was used to acquire 3D T1 turbo field echo (TFE) MRI data from all participants using the following imaging parameters: sagittal slice thickness, 1.0 mm; over contiguous slices with 50% overlap; no gap; repetition time, 9.9 ms; echo time, 4.6 ms; flip angle, 8°; and matrix size of 240 × 240 pixels reconstructed to 480 × 480 over a field of view of 240 mm to enhance the quality of images.

### Assessment of Lacunes on MRI

Lacunes were defined as small lesions ( ≤ 15 mm and ≥3 mm in diameter) with low signal on T1-weighted images, high signal on T2-weighted images, and a perilesional halo on 80 axial slices of FLAIR images (29). Two neurologists manually counted the number of lacunes, with a kappa value of 0.78.

### Volume Measurement of Subcortical Structures

FreeSurfer version 5.1.0 was used for the reconstruction of all 3D MR images (http://surfer.nmr.mgh.harvard.edu) in order to obtain the anatomical parcellations of subcortical structures. After parcellation, the labeled images were converted to the native anatomical space of the input MR data. The subcortical mesh surfaces were extracted from the labeled images for each subject by employing a Laplacian-based surface modeling system (30). Surface-based registration was performed by adopting a method described previously (31) to provide vertex correspondences across populations for all subcortical surface meshes. As mentioned above, 10 patients were excluded because of technical errors in this step.

### Follow-Up Evaluations With Neuropsychological Tests, MRI, and PiB-PET

About 101 patients with MCI underwent clinical interviews, neurological examination, neuropsychological tests, brain MRI, and PiB-PET imaging at baseline. Patients were annually evaluated for 3 years using clinical interviews, neuropsychological tests, and brain MRI. PiB-PET was followed-up in the second or third year. Of the 101 MCI patients, 95 completed the first year of clinical follow-up, 82 completed the second year of follow-up, and 83 completed the third year of follow-up.

Clinical follow-up for progression to dementia was performed until April 2015. We checked if patients had progressed to dementia during the clinical follow-up. The diagnosis of dementia was based on the criteria from the Diagnostic and Statistical Manual of Mental Disorders (fourth edition) and required evidence of impairment in social or occupational functions confirmed by the Seoul Instrumental ADL. For the diagnosis of probable AD and subcortical vascular dementia, we applied standard research criteria for dementia syndromes (32, 33).

**Figure 1:**
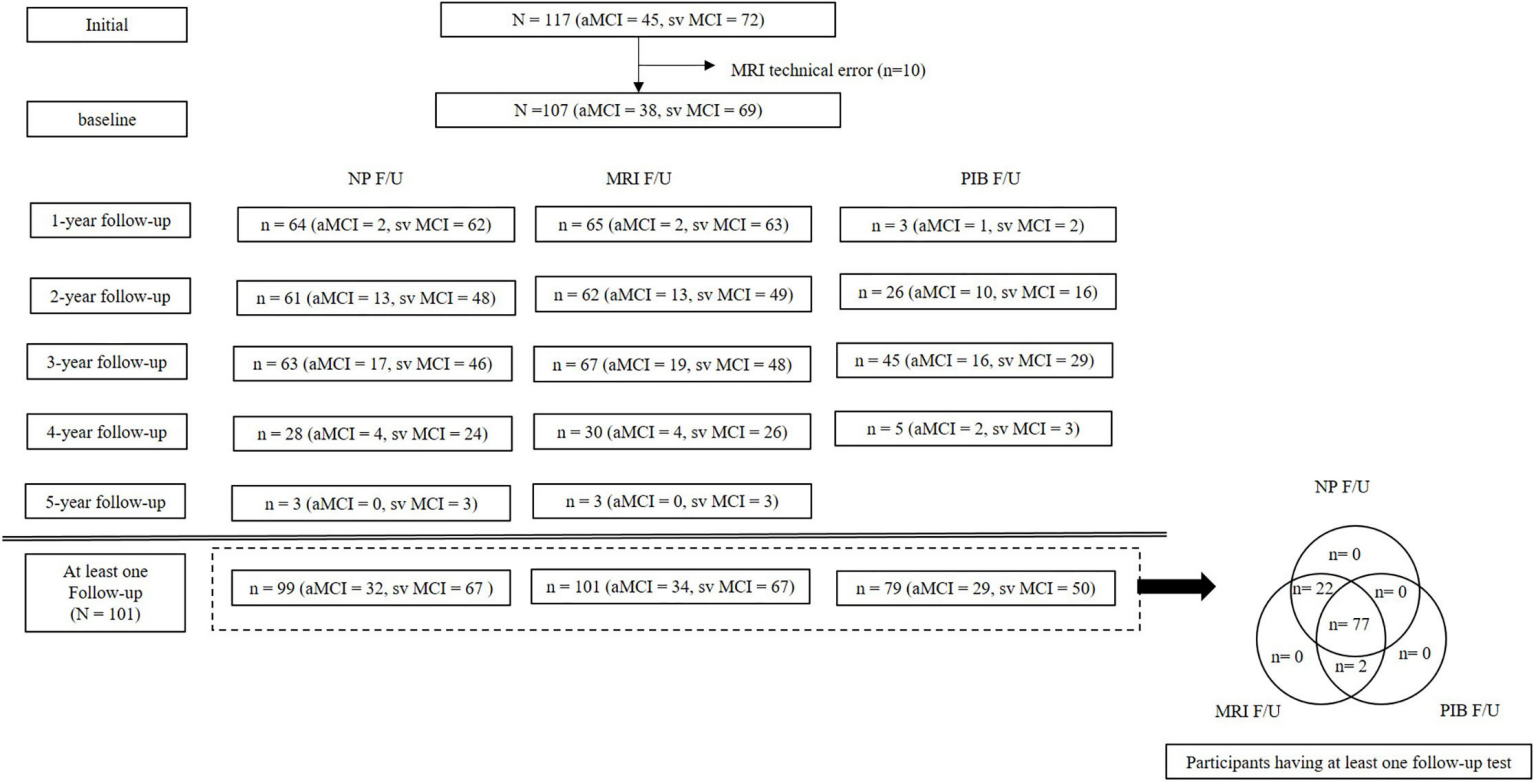
Participant flow diagram. MRI, Magnetic resonance imaging; NP, neuropsychological tests; PIB, Pittsburgh compound B.

In total, 99 patients had at least one follow-up neuropsychological test. Thirty-four patients with aMCI and 67 patients with svMCI had at least one follow-up brain MRI. The mean interval between the last follow-up MRI and baseline was 2.8 years for aMCI and 3.2 years for svMCI. Twenty-nine patients with aMCI and 50 patients with svMCI had follow-up PiB-PET scans. The mean interval between the follow-up PIB-PET and baseline was 2.7 years for aMCI and 2.7 years for svMCI. The number of participants at each annual testing time point is shown in Figure 1.

### Statistical Analyses

We used the paired *t*-test to estimate the difference between initial and follow-up values of PiB SUVR, which was normally distributed. Because the difference between the initial and follow-up number of lacunes was not normally distributed in the Shapiro-Wilk test, we performed the Wilcoxon signed-rank test instead. In the paired *t*-test, follow-up values of PiB SUVR and the number of lacunes were from the last performance of each test.

To obtain a wide range of values for PiB SUVR and the number of lacunes, we combined the two MCI subtypes (amnestic and subcortical vascular MCI) in the analyses, with the MCI subtype adjusted in each model. There was no multicollinearity in MCI subtypes and time-varying PiB SUVR or time-varying number of lacunes (variance inflation factor, VIF 1.304 for PiB; 1.402 for lacune). To investigate longitudinal changes in the volumes of subcortical structures according to time-varying PiB and lacunes, we performed linear mixed effects modeling including time-varying PiB SUVR and lacunes as independent variables, subcortical volume as the dependent variable, and baseline age, sex, years of education, intracranial volume (ICV), MCI subtypes, and time as covariates (random slope and intercept). Time-varying, here, means that the raw values at the given time points. We used the raw values of SUVR, lacune number, and cognitive scores in all analyses. Because the volume of subcortical structures decreased as the disease progressed, we used a covariance pattern for repeated time points as an autoregressive correlation structure.

When we selected subcortical structural regions to be included in the mediation analyses, we set the cutoff for significance with *p* < 0.05. To examine the mediation effect of subcortical structure on the association of PiB or lacunes with cognitive function, we performed linear mixed effects modeling instead of structural equation models or path analysis because mediators (subcortical structure), independent variables (PiB SUVR/number of lacune), and outcome (cognitive scores) were longitudinal data (34, 35). The model was composed of 3 steps. The first step was an association between time-varying PiB or lacunes and time-varying subcortical structures. The second step was the association between time-varying subcortical structures and time-varying cognition. The mediation effect (indirect effect) was calculated by multiplication of associations from step 1 and step 2. The third step was the association between time-varying PiB or lacunes and time-varying cognition. The coefficients from the third step represented the direct effect. The *p*-value of each step was computed for a bootstrap distribution (1,000 repetitions). We performed Bonferroni correction for comparisons across three cognitive scores, subcortical structures, and two linear mixed effects models. We adjusted the models for time, baseline age, sex, years of education, ICV, and MCI subtypes. Statistical analyses were conducted with R Version 4.0.2 lm4 Package.

## Results

### Demographic and Clinical Characteristics

Among 101 patients who completed at least one follow-up, 29 patients (28.7%) converted to dementia for 3 years. Specifically, 16/34 (47.0%) among patients with aMCI and 13/67 (19.4%) among patients with svMCI. Demographic and clinical characteristics are shown in Table 1.

**Table 1:** Demographic and clinical characteristics of the study participants.

	**Total**	**aMCI**	**Subcortical vascular MCI**	**p (aMCI vs. svMCI)**
N	101	34	67	-
Baseline age, years	72.9 ± 7.4	71.2 ± 8.1	73.8 ± 7.0	0.111
Sex, female (%)	58 (57.4)	16 (47.1)	42 (62.7)	0.133
Education, years	10.0 ± 5.2	12.4 ± 4.6	8.8 ± 5.0	0.001
*APOE4* carrier (%)*	31 (30.7)	12 (37.5)	19 (28.4)	0.359
Baseline PiB uptake	1.59 ± 0.46	1.80 ± 0.52	1.48 ± 0.38	0.001
Follow-up PiB uptake	1.65 ± 0.52	1.93 ± 0.55	1.50 ± 0.42	-
Baseline lacune number	4.7 ± 6.8	0.8 ± 2.5	6.7 ± 7.5	<0.001
Follow-up lacune number	5.1 ± 7.2	0.9 ± 2.9	7.3 ± 7.8	-
Follow-up duration, years	3.0 ± 1.0	2.8 ± 0.6	3.1 ± 1.1	-
Progression to dementia (%)	29 (28.7)	16 (47.0)	13 (19.4)	-
Cardiovascular risk factors				
Hypertension	66 (65.3)	15 (44.1)	51 (76.1)	0.001
Diabetes	22 (21.8)	5 (14.7)	17 (25.4)	0.22
Hyperlipidemia	28 (27.7)	5 (14.7)	23 (34.3)	0.037
Cardiac disease	20 (19.8)	6 (17.6)	14 (20.9)	0.699
Stroke	13 (12.9)	2 (5.9)	11 (16.4)	0.209
Baseline neuropsychological tests			
Memory composite score	53.0 ± 18.3	47.0 ± 14.2	56.0 ± 19.4	0.009
Executive composite score	31.5 ± 8.9	34.9 ± 9.6	29.6 ± 8.1	0.009
MMSE	26.2 ± 2.7	26.2 ± 2.1	26.3 ± 2.9	0.833
CDR-SOB	1.4 ± 0.9	1.6 ± 1.1	1.3 ± 0.8	0.145

Values are expressed as the mean ± SD or number (%). *Among 101 patients, 99 patients were examined using Apolipoprotein E (APOE) genotyping. aMCI, amnestic mild cognitive impairment; APOE, Apolipoprotein E; CDR-SOB, Clinical dementia rating scale sum of box; N, Number; PiB, Pittsburgh compound B; svMCI, subcortical vascular mild cognitive impairment.

In the follow-up PiB PET, 17 (59%) of 29 patients with aMCI and 30 (60%) of 50 patients with svMCI had elevated amyloid uptake compared to that on baseline PiB PET. PiB SUVR increased from 1.62 to 1.65 (*p* = 0.010, paired *t*-test in total patients; 1.88 to 1.91 in amnestic MCI and 1.46 to 1.50 in subcortical vascular MCI). The number of lacunes increased from 4.7 to 5.1 (*p* < 0.001, paired *t*-test in total patients; 0.8 to 0.9 in amnestic MCI and 6.7 to 7.3 in subcortical vascular MCI) after 3 years.

### Volume Changes in Subcortical Structure Related to Increased PiB SUVR and Number of Lacunes

Over three years, time varying PiB SUVR was associated with volume reduction in the amygdala (β = −0.2466, *p* = 0.0043) and hippocampus (β = −0.2698, *p* = 0.0039). Volume changes of caudate, pallidum, putamen, and thalamus were not associated with time varying PiB SUVR. Time varying lacune number was associated with volume reduction in the thalamus (β = −0.2050, *p* = 0.039). Volume changes of the amygdala, caudate, hippocampus, pallidum, and putamen were not associated with the time varying number of lacunes.

### Mediation Effect of Subcortical Volume Reduction in the Relationship Between PiB SUVR / Lacune and Cognitive Decline

**Figure 2:**
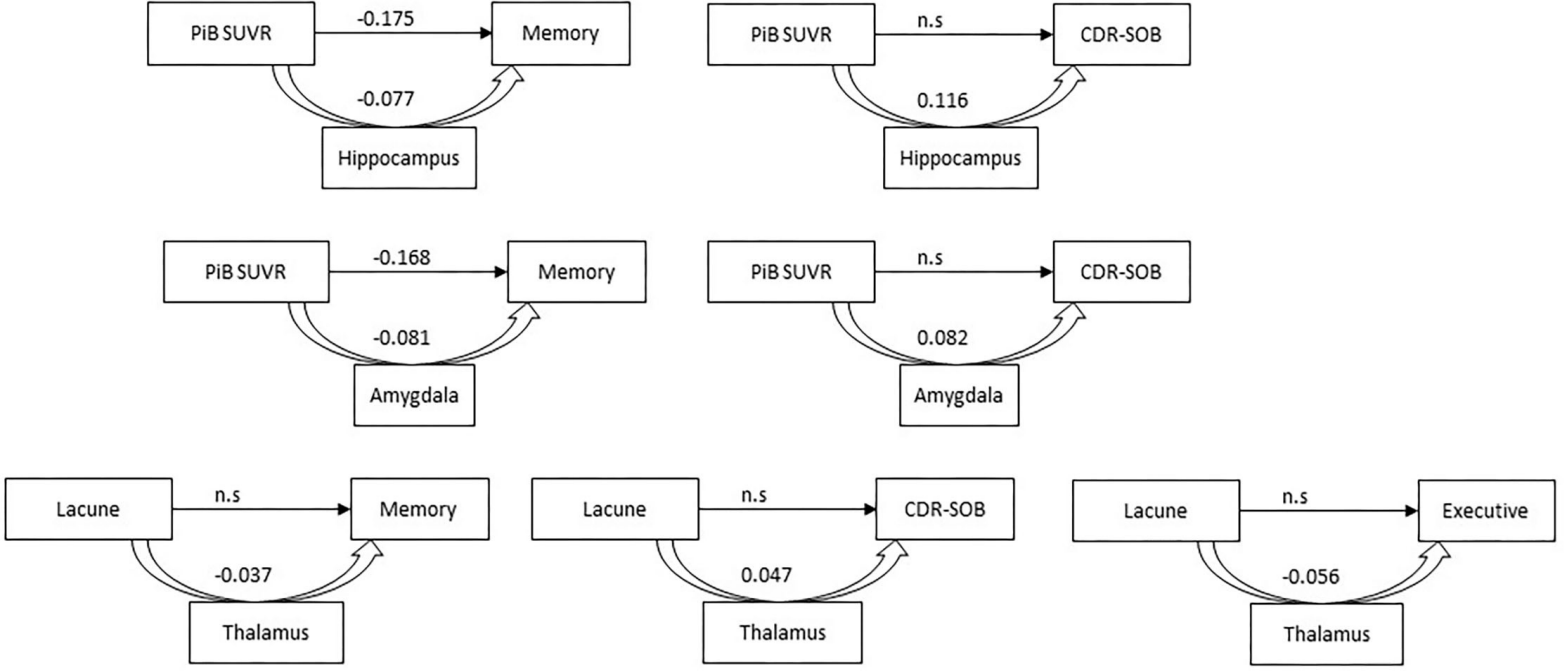
Schema of a mediation model. Arrows mean direct effect between Pittsburg compound B (PiB) standard uptake value ratio (SUVR)/lacune and cognition. Curved arrows signify an indirect effect of subcortical structures between PiB SUVR/lacune and cognition. CDR-SOB, dementia rating scale sum of box; n.s, no significant; PiB, Pittsburgh compound B; SUVR, Standard uptake value ratio.

We investigated the role of volume of amygdala and hippocampus, which had association with PiB SUVR, as mediators in the link between PiB SUVR and cognition. Figure 2 schematized the relationship. Amygdala and hippocampus partially mediated relationship between time-varying PiB and memory function [direct effect = −0.168 (*p* < 0.001), indirect effect = −0.081 (*p* < 0.001) for amygdala; direct effect = −0.175 (*p* < 0.001), indirect effect = −0.077 (*p* < 0.001) for hippocampus], and completely mediated relationship between time-varying PiB and CDR-SOB [indirect effect = 0.082 (*p* < 0.001) for amygdala; indirect effect = 0.116 (*p* < 0.001) for hippocampus]. There was no relationship between time varying PiB and frontal executive function (Table 2).

**Table 2:** Mediation analyses of subcortical structural volume changes in the relationship between Pittsburg compound B (PiB) standard uptake value ratio (SUVR)/lacune changes and cognitive decline.

**Dependent factor (cognition)**	**Mediator**	**Time-varying PiB SUVR**			
		**Estimate (A)**	**Adjusted p**	**Estimate (B)**	**Adjusted p**
**Memory**	Time-varying amygdalar volume	−0.168	<0.001	−0.081	<0.001
**Executive**		0.012	1.000	−0.026	0.468
**CDR-SOB**		0.207	0.072	0.082	<0.001
					
**Memory**	Time-varying hippocampal volume	−0.175	<0.001	−0.077	<0.001
**Executive**		−0.023	1.000	0.010	0.476
**CDR-SOB**		0.181	0.072	0.116	<0.001
		**Time-varying Lacune number**			
		**Estimate (A)**	**Adjusted p**	**Estimate (B)**	**Adjusted p**
**Memory**	Time-varying thalamic volume	0.063	0.468	−0.037	<0.001
**Executive**		−0.028	1.000	−0.056	<0.001
**CDR-SOB**		−0.011	1.000	0.047	<0.001

*Linear mixed effects modeling was used three times for mediation analyses: step 1, [time-varying PiB or lacunes] and [time-varying subcortical structures]; step 2, [time-varying subcortical structures] and [time-varying cognition]; step 3, [time-varying PiB or lacunes] and [time-varying cognition]. Estimate (A) was the association from step 3. Estimate (B) was calculated by multiplication of associations from step 1 and step 2. P-value (data not shown) was computed for a bootstrap distribution (1000 repetitions). Bonferroni correction was performed for comparisons across cognitive scores, subcortical structures, and two linear mixed effect models. We adjusted the models for time, baseline age, sex, years of education, intracranial volume (ICV), and mild cognitive impairment (MCI) subtypes. CDR-SOB, dementia rating scale sum of box; ICV, intracranial volume; MCI, Mild cognitive impairment; PiB, Pittsburgh compound B; SUVR, Standard uptake value ratio*.

On the link between the number of lacunes and cognition, we examined the role of volume of the thalamus, which was associated with the number of lacunes. Thalamus completely mediated relationship between time varying lacune number and cognitive function, including memory, frontal executive function, and CDR-SOB [indirect effect = −0.037, −0.056, and 0.047, respectively (*p* < 0.001)] (Table 2; Figure 2).

## Discussion

In the present study, we investigated the mediational effects of subcortical structures on the association of amyloid burden and lacunes with cognitive declines over 3 years in a cohort of carefully phenotyped patients with MCI using non-invasive amyloid imaging and structural MRI for markers of SVD. Our major findings were as follows. First, over three years of follow-up, volume reduction in the amygdala and hippocampus partially mediated the relationships between increased PiB SUVR and decline in the memory but not frontal executive function. Second, volume reduction in the thalamus completely mediated the relationships between increased lacune numbers and declines in both memory and frontal executive functions. Taken together, our findings provide a better understanding of the distinct role of subcortical structures in the mediation of the relationships of amyloid or vascular changes with a decline in specific cognitive domains.

Our first major finding was that, over 3 years of follow-up, volume reduction in the amygdala and hippocampus partially mediated the relationships between increased PiB SUVR and decline in the memory but not frontal executive function. Previous studies have shown that increased amyloid uptakes were associated with decreased hippocampal volume changes (36–38), which were further associated with memory decline (39). In addition, we also found that increased amyloid uptakes were associated with decreased amygdala volume changes which, in turn, leads to memory decline. Moreover, Aβ progression contributed to the decline of memory independently of the mediation effect of the amygdala and hippocampal volume changes. There are several possible explanations. Aβ may directly disconnect synapses, resulting in disruption of the functional network. Alternatively, Aβ progression might affect memory through another mechanism, such as neuroinflammation or thinning in the cortical regions related to memory decline (7).

Our second major finding was that an increased number of lacunes had only indirect effects on cognition mediated by volume reduction in the thalamus. Although previous studies showed increased lacune numbers are known to be associated with a decline in the frontal executive functions (7, 16), precise reasons were not revealed. We suggested that the mediative effect of the thalamus could be a key for the relationship between lacune and frontal executive functions. Thalamus is the main structure of frontal-subcortical circuits, which are responsible for frontal executive dysfunction (17). The pathobiology of the lacune effects on thalamic volume remains unclear. However, the development of lacunes in the thalamus may affect decreased thalamic volume changes because the thalamic region is vulnerable to developing lacunes (40, 41). Alternatively, lacunes might disrupt the thalamocortical connectivity, which in turn leads to decreased thalamic volume through secondary degeneration, such as Wallerian degeneration or dying back phenomenon (42).

Another noteworthy finding was that volume reduction in the thalamus mediated the relationships between increased lacune numbers and memory decline. Thalamus has been known to relate to the process of episodic memory and frontal executive function (43). The thalamus is the main structure of Papez's circuit (44) and dorsolateral limbic circuit (45) which are responsible for memory impairments. In fact, previous studies suggested that acute infarction in the thalamus affected memory impairments (46, 47). In addition, the thalamic volume changes were reported to be associated with memory impairments (48).

In the present study, we also found that volume changes in the amygdala, hippocampus, and thalamus completely mediated the relationships between increased amyloid or lacune numbers and worsening the CDR-SOB score. Our findings suggested that these subcortical structures affected global dementia severity because CDR-SOB is widely used to evaluate global dementia severity. CDR-SOB includes six domains of cognitive and functional performance applicable to AD and related dementias, namely, memory, orientation, judgment and problem solving, community affairs, home and hobbies, and personal care. Our findings might be explained by the fact that cognitive domains are related to memory and frontal executive dysfunction, which are also responsible for functional performance. Thus, our findings suggested that more attention to subcortical structures is needed to improve patients' symptoms. The strengths of our study are its longitudinal and prospective setting and standardized MRI imaging protocols. However, there are several limitations. First, we could not consider the effects of other neurodegenerative pathologies, including NFT, α-synuclein, transactive response DNA-binding protein, argyrophilic grain pathology, and hippocampal sclerosis because we performed the autopsy in only a few cases. Second, we did not examine WMH volume as a marker of SVD burden. We encountered some technical problems in measuring longitudinal changes in WMH. Specifically, some patients had a baseline WMH volume greater than the follow-up WMH volume. Third, it is possible that a learning effect will affect the cognitive function tests to some degree. Finally, our study population included a large proportion of cognitively impaired patients, which may limit the generalizability of our findings to other populations.

In conclusion, volume reduction in the amygdala and hippocampus partially mediated the relationships between increased amyloid burden and decline in the memory function, whereas volume reduction in the thalamus completely mediated the relationships between increased lacune numbers and declines in both memory and frontal executive functions. Therefore, our findings provide a better understanding of the distinct role of subcortical structures in the mediation of the relationships of amyloid or vascular changes with a decline in specific cognitive domains.

## Data Availability Statement

The raw data supporting the conclusions of this article will be made available by the authors, without undue reservation.

## Ethics Statement

The studies involving human participants were reviewed and approved by Institutional Review Board of Samsung Medical Center. The patients/participants provided their written informed consent to participate in this study.

## Author Contributions

N-YJ, J-HS, J-KS, and SS contributed to conceptualization and methodology and wrote sections of the manuscript. N-YJ wrote the first draft of the manuscript. HK, HJ, SM, SJK, YK, SC, KK, JK, YJ, STK, E-JK, and DN contributed to investigation and data collection. JV contributed to writing and revision. SL performed the statistical analysis. All authors contributed to manuscript revision, read, and approved the submitted version.

## Funding

This research was supported by a grant of the Korea Health Technology R&D Project through the Korea Health Industry Development Institute (KHIDI), funded by the Ministry of Health and Welfare and Ministry of Science and ICT, Republic of Korea (Grant Number: HU20C0111); a fund (2021-ER1006-00) by Research of Korea Disease Control and Prevention Agency; the National Research Foundation of Korea (NRF) grant funded by the Korea government (MSIT) (NRF-2019R1A5A2027340); Institute of Information & communications Technology Planning & Evaluation (IITP) grant funded by the Korea government (MSIT) (No.2021-0-02068, Artificial Intelligence Innovation Hub); a 2021 research grant from Pusan National University Yangsan Hospital; Brain Research Program through the National Research Foundation of Korea (NRF) funded by the Ministry of Science and ICT (No. 2020M3C7A101835721); the National Research Foundation of Korea (NRF) grant funded by the Korea government (MSIP) (No. 2019R1A2C109021211).

## Conflict of Interest

The authors declare that the research was conducted in the absence of any commercial or financial relationships that could be construed as a potential conflict of interest.

## Publisher's Note

All claims expressed in this article are solely those of the authors and do not necessarily represent those of their affiliated organizations, or those of the publisher, the editors and the reviewers. Any product that may be evaluated in this article, or claim that may be made by its manufacturer, is not guaranteed or endorsed by the publisher.
